# Factors Related to Adolescents’ Participation in Organized Sports

**DOI:** 10.3390/ijerph192315872

**Published:** 2022-11-29

**Authors:** Gwennyth E. Spruijtenburg, Femke van Abswoude, Sebastiaan Platvoet, Mark de Niet, Hidde Bekhuis, Bert Steenbergen

**Affiliations:** 1 Behavioural Science Institute (BSI), Radboud University, 6525 GD Nijmegen, The Netherlands; 2 Institute for Studies in Sports and Exercise, HAN University of Applied Sciences, 6525 GD Nijmegen, The Netherlands

**Keywords:** sport, adolescents, motor skills, motives, perceived competence, encouragement

## Abstract

Participation in organized sports has important health benefits in adolescence, yet participation rates are concerning. A better understanding of factors influencing adolescents’ participation in organized sports offers opportunities to improve participation rates. The aim of this study was to examine to what extent motives, perceived competence, encouragement and motor skills were associated with participation in organized sports in a sample of first-year secondary school students. In this cross-sectional study, data were collected from 386 Dutch adolescents (11–14 years) in October and November 2020. A series of multilevel logistic regression models estimated the likelihood of adolescents’ participating in organized sports. First, we examined whether motives, perceived competence, encouragement and motor skills were independently associated with the likelihood of participation. Second, we examined whether these factors were concurrently associated with the likelihood of participation. When considered individually, motives, perceived competence, encouragement and motor skills were significantly associated with participation. When considered together, only motives were significantly associated with participation and the associations of all other factors with sport disappeared. These findings show the importance of motivation for participation in sports over other factors. These findings inform the development of interventions aimed at improving adolescents’ participation rates.

## 1. Introduction

Participation in organized sports during adolescence positively influences physical, mental and psychosocial health and may also contribute to the development of an active adult lifestyle [1]. Despite these potential benefits, over a third of school-aged children do not participate in organized sports and their participation rates decline with age [2,3,4]. In the Netherlands, for example, participation in organized sports peaks at around the age of 10–14 (63% participation) and subsequently declines between the ages of 15 and 19 (44% participation) [5]. Given the important benefits of participation in organized sport, interventions and programs need to be developed with the aim of improving the concerning youth participation rates. To this end, we need a holistic understanding of the factors that influence sport participation in youth. Although multiple factors have been identified in the literature, few studies have investigated how these collective factors interact. A necessary next step is to examine the combinations of factors that affect involvement in sports (e.g., motor competence, motives, social support).

The Youth Physical Activity Promotion (YPAP) model by Welk [6] offers a theoretical framework to study the influences on adolescents’ participation in organized sports from a socioecological perspective. The YPAP model categorizes the factors that affect participation into three constructs: predisposing, reinforcing and enabling. First, the predisposing construct represents adolescents’ perceptions and motivation towards physical activities such as sports and consists of two sub-constructs: “Am I able?” and “Is it worth it?”. The “Am I able?” sub-construct is associated with adolescents’ perceptions of their motor competence. The “Is it worth it?” sub-construct is associated with adolescents’ enjoyment of and interest in physical activities. Second, the reinforcing construct represents social influences (e.g., parent, friend, teacher and coach). Third, the enabling construct represents environmental variables, which includes but is not limited to having access to sports equipment and opportunities to practice, as well as the skills that allow adolescents to be physically active. According to the YPAP model, all three constructs may directly encourage or discourage adolescents’ participation, whereas the reinforcing and enabling constructs may also influence participation indirectly through the predisposing construct. With the aim of generating new insights into the factors that affect youth participation in sports, it is important that studies concurrently include factors among all constructs of the model.

Previous systematic reviews have identified several factors at the predisposing, reinforcing and enabling levels that contribute to adolescents’ participation in physical activity and sports. As an example, Crane and Temple [7] showed that a lack of enjoyment of sports and a lack of perceived competence were the key determinants of declining participation in organized sports, thereby supporting the significance of the predisposing construct. Evidence for a reinforcing factor was demonstrated in yet another review [8]. In particular, Mendonça et al. [8] found that adolescents who receive encouragement from parents, friends and family had higher levels of overall physical activity. As a third example, concerning the enabling construct, there is evidence to suggest that children with well-developed motor skills participate in more physical activity [9]. These systematic reviews all emphasize the beneficial effects of individual factors, but do not provide information on how their interaction contributes to adolescents’ participation in organized sports. As a necessary next step, we will therefore combine these factors, and as such, consider all the levels of the YPAP model.

It is surprising that, to date, there are only a few studies that have started from this multifactorial account in which factors at different socioecological levels are combined to distinguish between youth who participate in sport (i.e., sporters) and youth who do not participate in sport (i.e., non-sporters) [10,11,12,13,14]. Four studies showed that combinations of factors at different levels such as gender, ethnicity, socioeconomic status, perceived competence and parental physical activity predicted participation in organized sports [11,12,13,14]. Surprisingly, these studies did not consider motives, despite the fact that these are the most common factors associated with participation in organized sports. One study did consider enjoyment together with other socioecological factors and established that enjoyment, next to behavioral intention, predicted continued participation in organized sports over and above other socioecological factors [10]. This study, however, did not include motor skills as a correlate, while it is known from previous research that this is an important aspect of participating in organized sports [15].

In the present cross-sectional study, we examine a unique combination of factors based on the YPAP model (i.e., motives, perceived competence, encouragement and motor skills) in a large group of adolescents (aged 11 to 14, *n* = 386). We perform multilevel logistic regressions to establish whether independent factors or specific combinations of factors are associated with the likelihood of adolescents’ participating in organized sports. It is hypothesized that all factors will increase the likelihood of adolescents’ participating in organized sports when considered individually. Based on the YPAP model, we also expect that these factors interact with each other when considered concurrently. This specific combination of factors has not been studied before. Because previous studies have shown that lack of motivation and perceived competence are key contributors to dropout from organized sport, we expect that these factors will contribute to a larger extent to adolescents’ participation in organized sports compared to encouragement and motor skills.

## 2. Materials and Methods

### 2.1. Study Design

The cross-sectional analysis presented here is part of an ongoing longitudinal cohort study within a larger research program (TRansitions Into Active Living, TRIAL) that focuses on changes in physical activity behaviors during key life transitions. Secondary school students were enrolled in the longitudinal study in October 2020, shortly after they transitioned from elementary to secondary school. We used the data from this first data wave (October 2020) for the analyses presented here. This study was approved by the Ethics Committee of the Faculty of Social Sciences at Radboud University (ECSW-2020-107).

### 2.2. Participants

Eight secondary schools from two provinces in the eastern part of the Netherlands that are part of ongoing collaborations were approached for participation as a convenience sample. Physical education teachers at these schools were contacted via email and informed about the study. Five schools agreed to participate (62.5%). The other three schools refused due to time constrains. In these five schools, all first-year students (*n* = 1127) and their parents were invited to participate. They received an information letter and informed consent forms on paper and via email. As a result, the parents of 531 students (mean age 12.45 ± 0.47; 52.0% female) agreed to have their child participate in the study. Of these 531 students, a total of 386 students (50.8% female) with a mean age of 12.48 years (SD = 0.47; range 11.19–14.17 years) had complete data on the variables used for the current analysis. These 386 participants were enrolled in different educational paths: 17% in vocational education, 16% in vocational/technical education, 22% in vocational/technical/academic education, 26% in technical/academic education and 19% in academic education. The ethnicity proportions were: 83% native Dutch and 17% non-native Dutch.

### 2.3. Assessments

#### 2.3.1. Background Measures

Students’ age, gender, ethnicity and BMI were assessed. Students self-reported their age and gender. For ethnicity, we followed the definition of Statistics Netherlands. Students were asked to report their country of birth and the countries of birth of their parents. Based on this information, they were categorized as native Dutch or non-native Dutch. Trained research assistants measured adolescents’ height (Seca stadiometer) and weight (Seca scale). BMI was then calculated (kg/m^2^).

#### 2.3.2. Sport

Participation in organized sports (yes or no) was assessed with a single item: “Do you participate in organized sports?”. Organized sport was defined as any sport that can be practiced in sport clubs (e.g., soccer, tennis, etc.) and fitness centers and any other sport that is led by a trainer or coach and includes formal practice (e.g., boot camp).

#### 2.3.3. Motives

Participants responded to eight items on a 4-point Likert scale (1 = totally disagree; 4 = totally agree) that began with “Why do/would you participate in organized sports?”. Items were based on the validated Motives for Physical Activity Measure—Revised (MPAM-R) [16]. The MPAM-R covers five motives for participating in physical activities such as sports: enjoyment, social, competence/challenge, appearance and fitness. We included one item for the enjoyment (“it is a fun thing to do”), appearance (“to improve body shape”) and fitness (“to be healthy”) motives, two items for the social motive (“to meet up with peers” and “friends participate too”) and three items for the competence/challenge motive (“to perform well”, “to be the best”, “to compete with others”). The questions were derived from previous studies on sport participation and physical activity among adolescents [17,18]. Exploratory factor analysis showed that, according to the Guttman–Kaiser rule [19], the different items form one factor. Therefore, motivation was calculated as the average of an individual’s responses to the eight items, with a Cronbach alpha of 0.76 indicating an acceptable level of reliability.

#### 2.3.4. Perceived Competence

Perceived competence was represented with a single item (based on the study of Best et al. [20]), “How good do you think you are at sports?”, on a 4-point Likert scale (1 = not good at all; 4 = very good).

#### 2.3.5. Encouragement

Six items were used to assess encouragement from father, mother, siblings, friends, trainer and physical education teacher, respectively: “How often does … encourage you to participate in sports?”. Again, a 4-point Likert scale was used (1 = never/almost never; 2 = almost always/always). Encouragement was calculated as the average of an individual’s responses on the six items, with a Cronbach alpha of 0.87 indicating an acceptable level of reliability.

#### 2.3.6. Motor Skills

Participants completed four motor skills tests: three test items of the KTK short form [21] (i.e., walking backwards, moving sideways and jumping sideways) and Faber’s [22] eye–hand coordination test. This combination of tests, which has recently been proposed by Platvoet et al. [23] and validated by Coppens et al. [24], objectively assesses fundamental movement skills performance. All motor skills competence tests were assessed shoeless and in light clothing. To obtain one overall score, a factor analysis with varimax rotation was conducted on the raw scores to compute the overall factor score for motor skills.

### 2.4. Procedures

Data collection procedures were carried out during a regular physical education class in an indoor facility by a team of trained testers. During the visit, participants completed an online questionnaire on their laptop computers or smartphone via LimeSurvey. The questionnaire assessed participation in organized sports, motives, perceived competence, encouragement, age and ethnicity. In addition, during the same visit, four motor skills tests were administered, and anthropometric measurements were taken.

### 2.5. Data Analysis

Age, BMI, motives, perceived competence, encouragement and motor skills were centered around the mean. Descriptive statistics were calculated for all variables. To examine which factors were associated with adolescents’ participation in organized sports, we conducted a series of multilevel logistic regressions. We accounted for a two-level structure with adolescents nested in classes (*n* = 38). Although classes were also nested in schools (*n* = 5), the number of schools was too low to perform three-level analyses [25]. Participation in organized sports was dichotomic (0, 1), and, therefore, we used the multilevel logistic regression command ‘xtlogit’ with robust standard errors in STATA 15.1 to explain participation in organized sports. The conventional multilevel logistic regression model incorporates cluster-specific random effects to account for the within-cluster correlation of subject outcomes: logit(Pr(*Y_ij_* = 1)) = *α*_0_ + *α*_0*j*_ + *α*_1_*x*_1*ij*_ + ⋯ + *α_k_x_kij_* + *β*_1_*z*_1*j*_ + ⋯ + *β_m_z_mj_* where *α*_0*j*_~*N*(0, *τ*^2^). The assumption is made that the random effects are independent of the model covariates (X,Z) (Austin and Merlo [26]).

In model 1, we included all background measures and participation in organized sports. In models 2a–d, we examined which factors were individually associated with the likelihood of participating in organized sports by adding one factor at a time to model 1. The order in which factors were added in models 2a–d was based on the directions of direct and indirect effects of the YPAP model: motor skills, encouragement, perceived competence and motives, respectively. In order to examine the combined effects of the factors on the likelihood of participating in organized sports, in models 3–5, we simultaneously added factors to the basic model (model 1). In model 3, both motor skills and encouragement were added. Next, we added perceived competence (model 4) and finally motives were added (model 5). Again, this was based on the predictions from the YPAP model.

To examine whether motor skills, encouragement and perceived competence indirectly affected participation in organized sports, we performed a variant of the Sobel test in Stata [27], which allowed us to test two indirect effects simultaneously. Indirect effects were computed by using the product of the coefficients method, which determines the indirect effect by multiplying the regression coefficients. We added product terms for all indirect effects to calculate the total indirect effect. Standard errors and confidence intervals were corrected through bootstrapping (5000 replications). Specifically, we tested whether motor skills had an indirect effect via motives or perceived competence and whether encouragement also indirectly affected participation in organized sports via motives or perceived competence. The applied significance level in all analyses was *p* < 0.005.

## 3. Results

The descriptive statistics of all variables can be found in Table 1. Table 2 shows the results of the multilevel logistic regression analyses on participation in organized sports. Model 1 only included the control variables. It appeared that only age had a marginal effect on sport participation; the older a student was, the smaller the chance that they participated in organized sports (OR = 0.001). Note that age is measured in thousands of a year; this precision makes the age effect seem very large. It also appears that the intraclass correlation (Rho) is 0.022, meaning that the percentage of the total variance that accounts for the class is 2.2%.

In models 2a–d (see Table 2), we included the independent variables motor skills, encouragement, perceived competence and motives separately. All these variables increased the likelihood to participate in organized sports with a higher score on the variable leading to a higher chance participating in sports. The increase in the likelihood to participate in organized sports was the smallest for motor skills (OR 1.008), followed by encouragement (OR 4.007) and perceived competence (OR 80.706), and the increase in likelihood was the largest for motives (OR 1298.390).

Next, in model 3, we included motor skills and encouragement together (see Table 2). Although the effects of the individual variables became marginally smaller than in the previous model (motor skills OR 1.007, encouragement OR 3.384), it was evident that students scoring higher on both variables had a higher likelihood to participate in organized sports.

In model 4, we added perceived competence to the variables of model 3 (see Table 2). The effect of perceived competence was positive, meaning that a higher score increased the likelihood to participate in organized sports (OR 38.863). The effect of encouragement remained largely unchanged and the influence of motor skills was no longer significant (*p* = 0.032).

In model 5, we added the variable motives (see Table 2). Motives had a large effect on the likelihood to participate in organized sports (OR 224.331), although smaller than the individual effect of motives found in model 2d. Importantly, adding motives to the model resulted in decreased effects of the other variables, none of which had a statistically significant effect on the likelihood of participating in organized sports.

Finally, we tested the indirect effects of encouragement and motor skills via perceived competence or motivation using the product of the coefficients method. Although the indirect effects were in the expected direction (i.e., motor skills had a positive influence on motives*,* which positively influenced sports participation), none of these effects were statistically significant.

## 4. Discussion

This paper adds to the existing literature on youth participation in sports by examining the extent to which a unique set of factors, i.e., motives, perceived competence, encouragement and motor skills, were associated with the likelihood of adolescents’ participating in organized sports. Corroborating earlier studies, we showed positive, independent effects of motives, perceived competence, encouragement and motor skills on participation in organized sports. As an extension of these earlier studies, we showed that, when taking all these factors together, the effect of motives outweighed the effects of the other factors. Below, we will discuss these findings in more detail.

First, the results of our study showed that motives, perceived competence, encouragement and motor skills each individually related to a higher likelihood of adolescents’ participating in organized sports. This finding is in agreement with previous studies as is shown in various systematic reviews [7,8,9]. These reviews showed persistent evidence for similar independent relationships between these factors and either overall physical activity or dropout from organized sports. These independent relationships were furthermore confirmed across different research designs in both children and adolescents. However, none of the relationships were identified for adolescents’ participation in organized sports. Both encouragement [8] and motor skills [9] have been related to overall physical activity, and motives and perceived competence have been related to dropout from organized sports [7]. In addition, no studies have been found in which motives, perceived competence, encouragement as well as motor skills were studied concurrently in the same population. Therefore, our results do not only corroborate previous findings, but also show that these four factors relate to participation in organized sports in a sample of first-year secondary school pupils.

Second, our results showed that motives for sport outweigh the effects of all other factors. That is, when considered together, the only factor that significantly related to a higher likelihood of adolescents’ participating in organized sports was motives. These findings seem to contradict the socioecological perspective of the YPAP model which suggests that factors within the predisposing, reinforcing and enabling constructs would all affect participation in organized sports collectively. Our results, however, suggest that when analyzing these factors together in one model, they do differ in their relative importance for participation in organized sports with motives being the most important factor. The importance of motives was previously shown in a study by Gardner et al. [10]. They investigated whether enjoyment and intentions to continue sports predicted dropout from organized sports over and above other factors. Enjoyment and intentions to continue sports are closely related to the motives assessed in our study. In the study of Gardner et al. [10], motives were shown to be of more importance than demographic, individual and social factors (e.g., age, perceived competence, parental support). Taken together with our results it is evident that motivation towards sports is the most important factor for participation in organized sports.

Given the importance of motives, a crucial next step would be to unravel the common denominators of motivation, and which of these most strongly contribute to continued participation in organized sports. This will also inform how motives can be enhanced. In our study, we asked participants to score themselves on questions that are related to their motives for participating in sports (i.e., enjoyment, social, competence/challenge, appearance and fitness). All these factors have been shown in other studies to be associated with participation in sports and physical activity [16,18,28]. Preliminary work on the contribution of different motives for continued participation in sports was undertaken by Ryan et al. [16]. They showed that large individual differences exist in the relative strength of their motives for sports participation. Given these findings, future studies with adolescents that also explore the sources of motivation and their relative strength in more depth are warranted. Additionally, it is also relevant to study how motivation is influenced by the other factors such as motor competence, perceived competence and encouragement by significant others and how motives are formed or change over time. Does, for example, a shift from one class to another, or a change in PE teacher, influence adolescents’ feelings of encouragement or competence? Additionally, does this result in a change in their motivation to participate? The possibility of changing motives over time also implies that, in future studies, we should investigate whether these processes can be influenced through contextual factors. Related to this, Carn and Cothran [29] showed that social factors such as being able to connect with a coach and having social opportunities are a key component of enjoyment in physical education. It is reasonable to assume that changes in the sport or PE environment are perceived differently by individual adolescents. For future research, we suggest that longitudinal studies with a unique, individualized approach are warranted to provide a more in-depth understanding of how factors associated with participation in organized sports influence adolescents’ motives for staying involved in sports in the long run.

An unanticipated finding of our study was the high percentage of adolescents who participated in organized sports. While national data indicate participation rates of 63% [5], in our sample the participation rate was 87%. This difference might, in part, be explained by the characteristics of the schools that agreed to participate in our study. Three of the five participating schools pay special attention to sports activities in their curriculum. This focus on sports may form a bias in the present population as this focus especially attracts children that participate in organized sports and deters children that do not already participate in sports. In addition, we should mention that at the time of data collection, new COVID-19 restrictions came in place in the Netherlands, which also affected sport for children. Although outside sports without official competition for children was often possible, even this was forbidden for a short period. We acknowledge that this may influence sport participation and motives. However, given the high participation rate in this sample, the high scores on motives for sport and the fact that the COVID-19 restrictions affected everyone, we are confident that the influence on the current results is minimal.

## 5. Conclusions

This study contributes to our understanding of youth participation in sports. We replicated findings from several earlier studies by showing that motives, perceived competence, encouragement and motor skills were each individually associated with an increased likelihood of adolescents’ participating in organized sports. We extended previous findings by showing that motives for sport were clearly the most important factor for sports practice when collectively analyzed. We suggest that the other factors (i.e., perceived competence, encouragement and motor skills) may influence and precede motives for sports participation. In line with this, further research is warranted to understand the causal relationships between these factors, motivation and sport participation. To our knowledge, this is the first study to show that motives are more important than perceived competence, encouragement and motor skills in predicting the likelihood of adolescents’ participating in organized sports. The findings of this study indicate that, in order to develop effective programs for stimulating young people to continue sport participation, maintaining motivation towards sports should be the main priority of youth sport organizations.

## Figures and Tables

**Table 1:** Descriptive statistics after listwise deletion before centering to the mean.

		%	Mean	Std. Dev.	Range
Sport	Yes	87.300			
	No	12.700			
Motor skills			0.010	0.804	−3.017–2.367
Encouragement			1.971	0.855	1–4
Perceived competence			3.251	0.600	1–4
Motives			3.331	0.503	1.375–4
Gender	Boy	49.200			
	Girl	50.800			
Age			12.482	0.472	11.190–14.174
Ethnicity	Native Dutch	83.160			
	Non-native-Dutch	16.840			
BMI			18.268	2.495	13.316–29.702
*n* = 386					

**Table 2:** Multilevel multivariate logistic regression analyses on sport participation.

	Model 1			Model 2a			Model 2b			Model 2c			Model 2d			Model 3			Model 4			Model 5		
	OR	95% CI		OR	95% CI		OR	95% CI		OR	95% CI		OR	95% CI		OR	95% CI		OR	95% CI		OR	95% CI	
Gender																								
Boy = ref.	1.170	0.647	2.116	1.273	0.678	2.392	1.158	0.615	2.182	0.879	0.469	1.646	0.769	0.381	1.552	1.211	0.627	2.338	0.975	0.504	1.889	0.813	0.395	1.672
Age	**0.001**	**0.000**	**0.610**	0.012	0.000	7.016	0.001	0.000	1.230	**0.000**	**0.000**	**0.086**	**0.000**	**0.000**	**0.036**	0.012	0.000	9.863	0.001	0.000	0.641	**0.000**	**0.000**	**0.046**
Ethnicity																								
Dutch = ref.	1.592	0.854	2.970	1.792	0.901	3.565	1.588	0.879	2.869	1.533	0.825	2.846	1.672	0.798	3.505	1.748	0.948	3.224	1.530	0.812	2.881	1.552	0.768	3.135
BMI	0.178	0.012	2.652	0.504	0.036	7.084	0.173	0.011	2.702	0.264	0.020	3.461	0.140	0.008	2.315	0.405	0.030	5.494	0.514	0.033	7.970	0.297	0.020	4.332
Motor skills				**1.008**	**1.004**	**1.013**										**1.007**	**1.003**	**1.012**	1.006	1.000	1.011	1.004	0.999	1.009
Encouragement							**4.007**	**2.287**	**7.023**							**3.384**	**2.010**	**5.696**	**2.940**	**1.609**	**5.374**	1.775	0.873	3.609
Perceived competence										**80.706**	**16.052**	**405.771**							**38.863**	**8.366**	**180.533**	5.648	1.166	27.354
Motives													**1298.390**	**63.401**	**26,589.830**							**224.331**	**9.479**	**5309.251**
Intra class correlation	0.022			<0.001			0.042			<0.001			<0.001			<0.001			0.002			<0.001		
Log pseudolikelihood	−142.627			−137.011			−136.637			−129.981			−118.325			−132.395			−124.206			−114.845		

Numbers in bold, sig. *p* < 0.005.

## Data Availability

The data presented in this study are openly available in DANS EASY at https://doi.org/10.17026/dans-255-ch4s.
